# The Effect of Combined Treatment of Psilocybin and Eugenol on Lipopolysaccharide-Induced Brain Inflammation in Mice

**DOI:** 10.3390/molecules28062624

**Published:** 2023-03-14

**Authors:** Timur Zanikov, Marta Gerasymchuk, Esmaeel Ghasemi Gojani, Gregory Ian Robinson, Shima Asghari, Alyssa Groves, Lucie Haselhorst, Sanjana Nandakumar, Cora Stahl, Mackenzie Cameron, Dongping Li, Rocio Rodriguez-Juarez, Alexandra Snelling, Darryl Hudson, Anna Fiselier, Olga Kovalchuk, Igor Kovalchuk

**Affiliations:** 1 Department of Biological Sciences, University of Lethbridge, Lethbridge, AB T1K 3M4, Canada(; 2 Institute for Medical Nutrition Science, Universität zu Lübeck, 23562 Lübeck, Germany; 3 School of Biosciences and Technology, Vellore Institute of Technology, Vellore 632014, India; 4 Department of Medicine, Medical Sciences, and Nutrition, University of Aberdeen, King’s College, Aberdeen AB24 3FX, UK; 5 GoodCap Pharmaceuticals, 520 3rd Avenue SW, Suite 1900, Calgary, AB T2P 0R3, Canada; 6 Department of Family Medicine, Cumming School of Medicine, University of Calgary, Calgary, AB T2N 4N1, Canada

**Keywords:** psilocybin, eugenol, LPS, inflammation, brain

## Abstract

Inflammation is an organism’s biological defense mechanism. Acute and chronic inflammation of the body triggers the production of pro- and anti-inflammatory pathways that can affect the content of cytokines in the brain and thus cause brain inflammation. Disorders such as depression and posttraumatic stress disorder (PTSD) are often associated with elevated inflammation. Recently, positive and promising clinical results of psilocybin for the treatment of depression and PTSD were reported. Thus, we decided to test whether psilocybin alone or in combination with eugenol, an anti-inflammatory and antioxidant agent, would prevent the increase in or decrease the content of cytokines in the brain of C57BL/6J mice injected with lipopolysaccharides (LPS). Two experiments were performed, one with pre-treatment of mice through gavage with psilocybin (0.88 mg/kg), eugenol (17.6 mg/kg), or combinations of psilocybin and eugenol (1:10, 1:20, or 1:50), followed by intraperitoneal injection of LPS, and the second, post-treatment, with initial injection with LPS, followed by treatment with psilocybin, eugenol, or their combination. Brain tissues were collected, and cytokines were analyzed by qRT-PCR, Western blot, and ELISA. Data were analyzed with a one-way ANOVA followed by Tukey’s post hoc test or with multiple unpaired t-tests. LPS upregulated mRNA expression of *COX-2*, *TNF-α*, *IL-1β*, and *IL-6*. All pre-treatments decreased the expression of *COX-2* and *TNF-α*, with psilocybin alone and in 1:50 combination, with eugenol being the most effective. In the post-treatment, all combinations of psilocybin and eugenol were effective in reducing inflammation, with the 1:50 ratio displaying the most prominent results in reducing the mRNA content of tested cytokines. Western blot analysis confirmed the effect on COX-2 and IL-1β proteins. Finally, the ELISA showed that post-treatment with psilocybin + eugenol (1:50) demonstrated the best results, decreasing the expression of multiple markers including IL-6 and IL-8. This demonstrates the anti-inflammatory effects of a combination of psilocybin and eugenol in the brain of animals with systemically induced inflammation.

## 1. Introduction

Neuroinflammation is an inflammatory response within the brain or spinal cord. Various factors can trigger neuroinflammation, including traumatic brain injury, infections, toxins and toxic metabolites, and immune dysregulation [1]. Multiple pro-inflammatory cytokines, chemokines, secondary messengers (NO and inositol trisphosphate), and reactive oxygen species (ROS) contribute to this inflammatory response. In the central nervous system (CNS), many of these mediators are produced by activated cells, such as microglia and astrocytes, endothelial cells, and immune cells derived from the peripheral nervous system [2,3].

Neuroinflammation protects the brain against insults by removing or inhibiting noxious agents and reversing their effects [4]. Additionally, the inflammatory response promotes tissue repair, supports the blood–brain barrier, and removes cellular debris that would otherwise contribute to neurodegeneration and disease progression. Early inflammation is vital to the healing and regeneration of tissue following some insults and can thereby lead to neurodegeneration [5,6].

There are various mechanisms by which neuroinflammation arises, depending on its underlying cause. During the inflammation that occurs after a concussion, pro-inflammatory cytokines are released, which can worsen the damage already caused by the physical injury and cause DNA fragmentation and cell death. The release of additional cytokines can also compromise the blood–brain barrier, which reduces its ability to prevent pathogens and other toxins from passing through it.

Both cognitive degeneration and neurodegenerative diseases are associated with inflammation and with aging. In a healthy, aging brain, pro-inflammatory cytokines are chronically increased, and anti-inflammatory cytokines are reduced. Moreover, research has revealed that the aging brains exhibit an increased number of activated microglia, indicating that the immune system is activated. Clearly, there is a link between neuroinflammation and the aging brain.

Inflammation is responsible for much of the neurodegeneration associated with Alzheimer’s disease. Microglia in AD are associated with amyloid plaque formation [7].

Gut inflammation is associated with the pathogenesis of Parkinson’s [8] and is thought to influence the brain, impacting the substantia nigra, thereby disrupting dopamine production and leading to disease progression.

Evidence suggests that inflammation plays a significant role in psychiatric illnesses as well. Brain inflammation has been linked to a wide range of diseases, including depression, schizophrenia, PTSD, and mood disorders [9].

In Gram-negative bacteria, the outer membrane contains large polysaccharide and lipid molecules known as lipopolysaccharides (LPS). Upon infection with Gram-negative bacteria, they are a major triggering factor for the inflammatory cascade. When LPS interact with their receptors, several intracellular molecules are activated that alter the expression of various inflammation-related mediators. As a result, neurodegenerative processes are triggered. Through the TLR-4 signaling pathway, LPS cause neuroinflammation, which results in cognitive impairment. This makes LPS valuable tools for the study of neuroinflammation in neurodegenerative diseases [10,11].

Eugenol, an aromatic compound commonly used as a topical pain reliever during dental procedures, is found in plants such as cloves, bay leaves, and allspice. Pharmacological studies have demonstrated that eugenol is an effective free radical scavenger with anticonvulsant, bactericidal, antifungal, analgesic, antiseptic, hepatoprotective, and antioxidant properties. The mechanism of eugenol’s anti-inflammatory action involves the inhibition of tumour necrosis factor α (TNF-α) and reduced production of nitrous oxide radicals [12,13,14].

In 2017, Said and Rabo examined the protective effects of eugenol supplementation against aluminum (Al)-induced neural damage in rats. Co-administration of Al and eugenol restored brain-derived neurotrophic factor (BDNF) and 5-HT (serotonin) levels and enhanced total antioxidant status (TAS) in the brain. Eugenol co-administration also decreased upregulated TNF-α expression [15].

Parween et al. showed that eugenol significantly improved healthy ageing and slowed neurodegeneration in a CL4176 worm (Caenorhabditis elegans) Alzheimer’s model by enhancing oxidative stress resistance and slowing paralysis [16].

Taheri et al. studied the effects of eugenol on an Alzheimer’s model in rats. Rats treated with 0.01 mg/kg eugenol showed improved memory and had a significant decrease in amyloid plaques [17].

Akbar et al. examined the effects of eugenol in healthy mice and found it improved hippocampal dendritic complexity and memory performance, increased neurogenesis, and decreased the number of apoptotic cells in the dentate gyrus and cornu ammonis 1 basal regions [18]. As reported by Revi and Rengan, eugenol had the ability to polarize microglia from a pro-inflammatory to an anti-inflammatory state [19]. Due to its antioxidant, anti-apoptotic, and neurotrophic properties, eugenol has strong potential to be a neuroprotective agent.

Psilocybin-containing mushrooms have been used for their healing properties throughout history. Most of the research on psilocybin has focused on its antidepressant properties [20,21]. Psilocybin’s success in treating various mental health disorders, such as anxiety, depression, and obsessive–compulsive disorder, has changed how it is perceived in the medical community.

Psilocybin is a 5-HT2A (serotonin) receptor agonist. Despite serotonin’s pro-inflammatory effects on 5-HT2A receptors, psilocybin has been shown to exert strong anti-inflammatory effects in animal models of inflammatory disorders [22,23]. Additionally, psychedelics have been shown in cell and animal models to inhibit inflammation induced by TNF-α.

There is great potential for psychedelics, psilocybin especially, to be used for treating neuroinflammatory disease. For example, all known genetic and environmental risk factors for AD are associated with inflammation, suggesting that reducing inflammation could be a target for disease prevention [24].

The administration of 5-HT1A and 5-HT2A receptor agonists to rats with streptozotocin-induced AD demonstrated significant neuroprotective effects in hippocampal neurons through anti-apoptotic and anti-inflammatory pathways [25]. In particular, activation of 5-HT2A receptors in rodent neurons increases Sirtuin 1 expression, which protects against reactive oxygen species as changes to SIRT1 expression and activity have been linked to inflammatory diseases [26,27]. Psilocybin and other psychedelics have been shown to stimulate neurogenesis, induce neuroplastic changes, and reduce neuroinflammation [28].

To determine whether psilocin has advantages for neural tissue homeostasis and promotes anti-inflammatory and regenerative effects, Kozkowska and colleagues examined its effects on activated microglia in a mouse model. Post-psilocin, pro-inflammatory proteins (TLR4, p65, and CD80) were downregulated, and TREM2, which is linked to neuroprotection and proper microglial phagocytosis, was upregulated. In addition, psilocin inhibited the phagocytosis of healthy neurons by microglia and caused a reduction in microglial pro-inflammatory responses [29]. It can be concluded that psilocybin is an effective therapeutic molecule for treating multiple neural conditions characterized by inflammatory pathogenic processes.

The potential for psychedelic compounds to influence and enhance functional neuronal connectivity, stimulate neurogenesis, restore brain plasticity, reduce inflammation, and enhance cognition provides a new therapeutic target and compelling argument for further investigation of the potential for psychedelics as a disease-modifying compound in conditions where currently none exists.

Psilocybin has the capacity to function as an antioxidant and may therefore be effective at reducing inflammation-induced oxidative stress. Despite its anti-inflammatory properties, psilocybin has not yet been extensively examined in terms of its effects on brain inflammation. In this study, we aimed to investigate whether the application of eugenol and psilocybin, both separately and together, would have anti-inflammatory features in murine brains. We tested the effects of eugenol and psilocybin on LPS-induced brain inflammation in mice.

## 2. Results

### 2.1. Induction of Inflammation with LPS

We injected mice (i.p.) with 0.83 mg/kg LPS to induce inflammation in the brain [30]. This dose was previously shown to be effective in stimulating the expression of pro-inflammatory cytokines in the periphery within 1–2 h of injection and in the brain within 3–4 h; the effect in the brain lasted for at least 24 h. We collected brains from animals at 4, 24, and 48 h post-injection to analyze cytokine content.

#### 2.1.1. Upregulation of Cytokines as Shown by qRT-PCR

**Figure 1:**
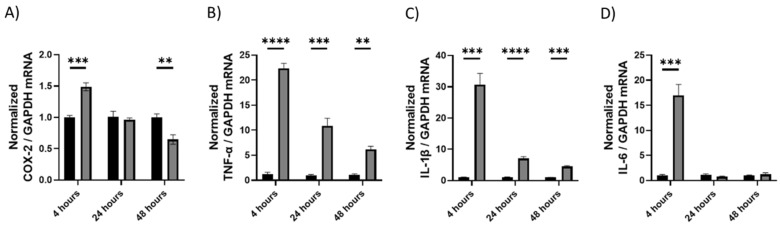
The effect of LPS on mRNA expression in brain tissue at 4, 24, and 48 h. Changes in mRNA expression as measured by RT-qPCR for: (**A**) *COX-2*, (**B**) *TNF-α*, (**C**) *IL-1β*, (**D**) *IL-6*. Glyceraldehyde-3-phosphate dehydrogenase (*GAPDH*) was used as a loading control. Data were analyzed with (**A**–**D**): Multiple t-tests were performed with a false discovery rate correction (Q = 5%, *n* = 3–6). Significance (*p*) is indicated within the figures using the following scale: **, *p* < 0.01; ***, *p* < 0.001; ****, *p* < 0.0001. Bars represent mean ± SEM.

mRNA was isolated from brain tissue and analyzed via RT-qPCR. The expression of pro-inflammatory cytokines and enzymes was quantified. All four cytokines were upregulated at 4h (*p* < 0.001). *TNF-α* and *IL-1β* were upregulated at all 3 time points (*p* < 0.001, Figure 1B,C, respectively), while *COX-2* was downregulated after 48 h (*p* < 0.01, Figure 1A).

#### 2.1.2. Changes in Cytokines Revealed by Western Blot

**Figure 2:**
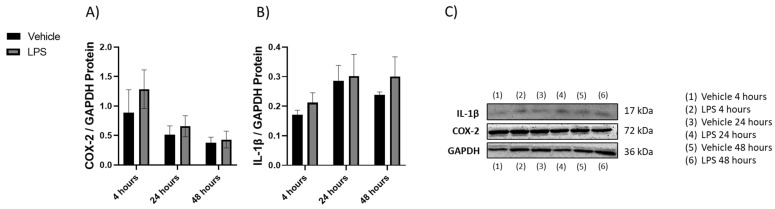
The effect of LPS and vehicle treatments on expression of COX-2 and IL-1β in 4, 24, and 48 h time points. Figures represent changed protein expression for selected genes measured by Western blot. Glyceraldehyde-3-phosphate dehydrogenase (GAPDH) was used as a loading control. Relative densitometry is presented as a ratio to GAPDH. (**A**) COX-2 expression, (**B**) IL-1β expression, (**C**) representative images blots with each protein detected. Original membranes can be seen in Figures S3–S5. Multiple *t*-tests were performed with a false discovery rate correction (Q = 5%, *n* = 3). The samples for each protein were run on the same gel. No image enhancements were applied.

Protein was isolated from brains collected from mice exposed to LPS, and the expression of COX-2 and IL-1β proteins was measured. No statistically significant changes were observed (Figure 2).

### 2.2. Pre-Treatment and Post-Treatment with Psilocybin and Eugenol

Based on the data from Figure 1, we decided to harvest tissues 24 h after LPS treatment; at that time point, the inflammation induced at 4 h persisted within the brain. We performed pre- and post-treatment experiments. In the pre-treatment experiment, we gave animals psilocybin, eugenol, or a combination at 48 and 24 h prior to injecting them with the LPS.

#### 2.2.1. Body Weight Measurements

**Figure 3:**
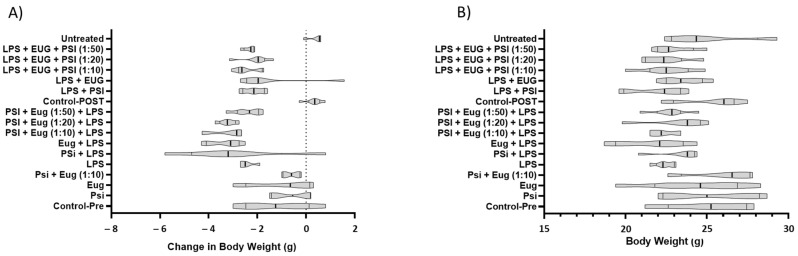
The effect of treatments on body weight (**A**) and body weight change (**B**) in the mouse model. Truncated violin plots. ANOVA and Tukey (*n* = 4–6). Significance of differences for (**A**) can be seen in Table S3.

There were no statistically significant differences in body weight at collection; however, statistically significant differences were noted in the change in body weight (Figure 3, Table S3). In the psilocybin + LPS pre-treatment group, mice gained up to 0.8 and lost up to 5.9 g of body weight. Notably, mice pre-treated with eugenol + LPS and mice pre-treated with psilocybin + eugenol (1:10) + LPS showed a similar decrease in weight between 2.5 to 4 g. LPS + eugenol showed better results for the post-treatment compared to the pre-treatment condition. Not only did this result in less severe loss, which was between 1.5 to 2.4 g, but some of the mice in the group gained between 0.8 and 1.7 g of weight.

#### 2.2.2. RT-qPCR Analysis of Cytokines in Pre- and Post-Treatment Animals

**Figure 4:**
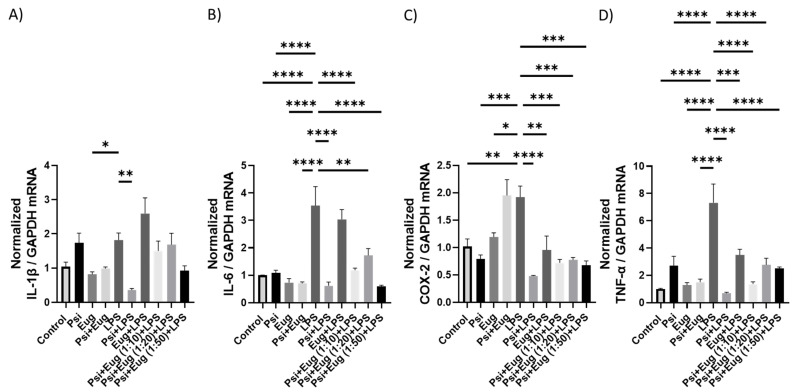
The effect of pre-treatment on mRNA expression in brain tissue. Changes in mRNA expression as measured by RT-qPCR for: (**A**) *IL-1β*, (**B**) *IL-6*, (**C**) *COX-2*, (**D**) *TNF-α*. Glyceraldehyde-3-phosphate dehydrogenase (*GAPDH*) was used as a housekeeping gene. Data were analyzed with ANOVA and Dunnett’s post hoc test compared to LPS (*n* = 3–6). Significance (*p*) is indicated within the figures using the following scale: *, *p* < 0.05; **, *p* < 0.01; ***, *p* < 0.001; ****, *p* < 0.0001. Bars represent mean ± SEM.

To understand the effects of psilocybin and eugenol as well as their combinations on brain inflammation, we analyzed mRNA expression by RT-qPCR. mRNA levels of *IL-1β* trended higher than the control (*p* = N.S.), while only the psilocybin pre-treatment (Psi + LPS) significantly reduced *IL-1β* compared to the LPS group (*p* < 0.01, Figure 4A). In contrast, *IL-6* mRNA levels were upregulated compared to the control (*p* < 0.0001), and downregulated by Psi + LPS (*p* < 0.0001), Psi + Eug (1:10) + LPS (*p* < 0.0001), Psi + Eug (1:20) + LPS (*p* < 0.0001), and Psi + Eug (1:50) + LPS (*p* < 0.0001) compared to the LPS group (Figure 4B). *COX-2* mRNA levels were significantly increased compared to the control (*p* < 0.01), while all pre-treatment groups significantly reduced relative *COX-2* levels (*p* < 0.01, Figure 4C). Interestingly, dual psilocybin and eugenol treatment resulted in similar relative *COX-2* levels to the LPS group (Figure 4C). Similar to *COX-2*, LPS significantly increased *TNF-α* mRNA levels compared to the control (*p* < 0.0001), while all pre-treatment groups significantly reduced *TNF-α* mRNA levels compared to the LPS group (*p* < 0.001, Figure 4D).

**Figure 5:**
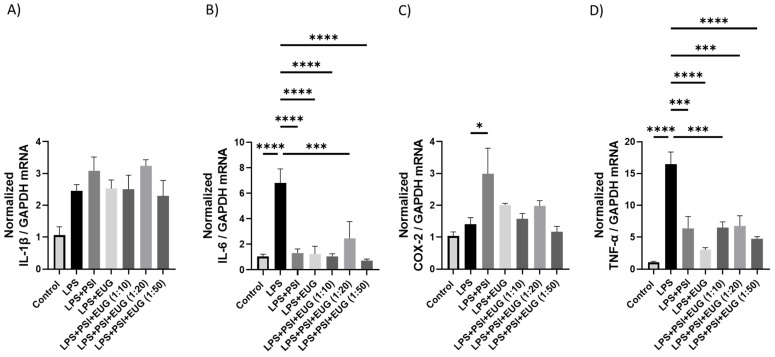
The effect of post-treatment on mRNA expression in brain tissue. Changes in mRNA expression as measured by RT-qPCR for: (**A**) *IL-1β*, (**B**) *IL-6*, (**C**) *COX2*, (**D**) *TNF-α*. Glyceraldehyde-3-phosphate dehydrogenase (*GAPDH*) was used as a housekeeping gene. Data were analyzed with ANOVA and Dunnett’s post hoc test compared to LPS (*n* = 3–6). Significance (*p*) is indicated within the figures using the following scale: *, *p* < 0.05; ***, *p* < 0.001; ****, *p* < 0.0001. Bars represent mean ± SEM.

While LPS treatment resulted in *IL-1β* levels that trended higher than the control (*p* = N.S.), no differences were seen in any post-treatment compared to the LPS group (*p* = N.S., Figure 5A). In contrast, relative *IL-6* mRNA levels were significantly upregulated in the LPS group compared to the control (*p* < 0.0001), and all post-treatment groups significantly downregulated *IL-6* mRNA levels compared to LPS group (*p* < 0.0001, Figure 5B). Surprisingly, LPS did not upregulate *COX-2* mRNA levels (*p* = N.S.), while LPS + Psi upregulated *COX-2* levels compared to the LPS group (*p* < 0.05, Figure 5C). Similarly, *TNF-α* was significantly upregulated in the LPS group compared to the control (*p* < 0.001), while all post-treatments significantly downregulated *TNF-α* levels compared to the LPS group (*p* < 0.001, Figure 5D).

#### 2.2.3. Protein Analysis of Pre- and Post-Treatment Groups by Western Blot

**Figure 6:**
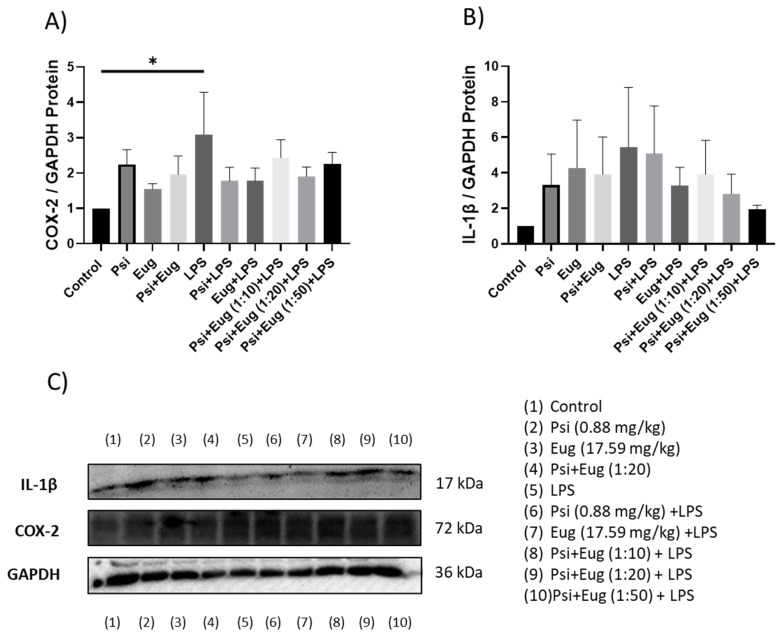
The effect of psilocybin and eugenol on the expression of (**A**) COX-2 and (**B**) IL-1β. (**C**) Representative images blots with each protein detected. Original membranes can be seen in Figures S6–S8. Figures represent changed protein expression for selected genes measured by Western blot in the pre-treatment group. Glyceraldehyde-3-phosphate dehydrogenase (GAPDH) was used as a loading control. Relative densitometry is presented as a ratio of the target protein to GAPDH. Data were analyzed with ANOVA and Dunnett’s post hoc test compared to LPS (*n* = 5–6). Significance (*p*) is indicated within the figures using the following scale: *, *p* < 0.05. Bars represent mean ± SEM.

Relative protein levels of COX-2 in the LPS group were shown to be upregulated compared to the control (*p* < 0.05, Figure 6A). While pre-treatment may lower COX-2 protein abundance, no significant changes were seen compared to the LPS group (*p* = N.S., Figure 6A). In contrast, IL-1β protein levels were not significantly altered across any of the pre-treatment groups; however, LPS appear to upregulate IL-1β levels (*p* = N.S.), while eugenol + LPS and combined treatment groups appear to decrease IL-1β levels compared to the LPS group (*p* = N.S., Figure 6B).

**Figure 7:**
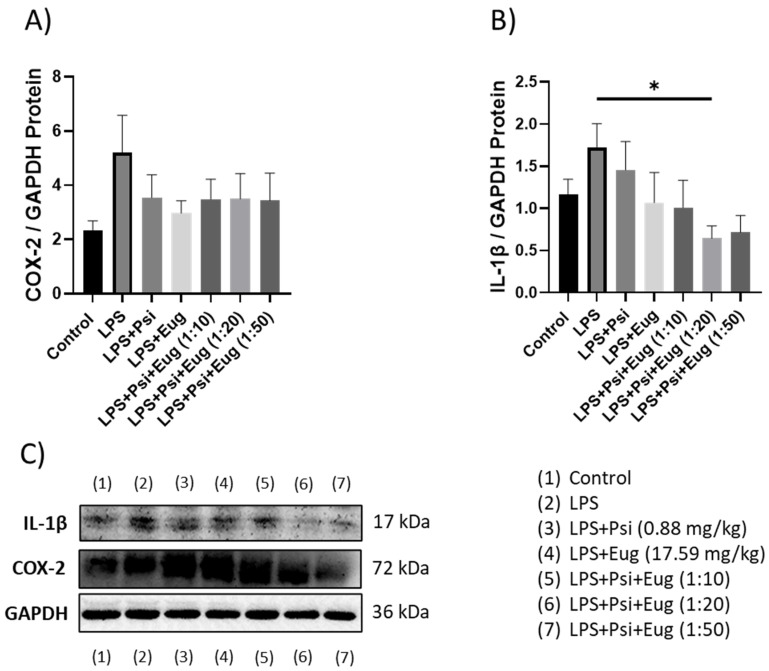
The effect of post-treatments on the expression of (**A**) COX-2 and (**B**) IL-1β. (**C**) Representative images blots with each protein detected. Original membranes can be seen in Figures S9–S11. Figures represent changed protein expression for selected genes measured by Western blot. Glyceraldehyde-3-phosphate dehydrogenase (GAPDH) was used as a loading control. Relative densitometry is presented as a ratio of target protein to GAPDH. Data were analyzed with ANOVA and Dunnett’s post hoc test compared to LPS (*n* = 5–6). Significance (*p*) is indicated within the figures using the following scale: *, *p* < 0.05. Bars represent mean ± SEM.

For post-treatment, both COX-2 and IL-1β levels appeared to be higher in the LPS groups compared to the controls (*p* = N.S., Figure 7A,B, respectively). While all treatments demonstrated a small trend to lower COX-2 protein levels, no significant differences were observed (*p* = N.S., Figure 7A). In contrast, the post-treatment group Psi + Eug (1:20) + LPS significantly lowered IL-1β levels compared to the LPS group (*p* < 0.05), while other psilocybin and eugenol post-treatment combination (1:10 and 1:50) groups showed a trend to lower IL-1β levels, but this was not significant (*p* = N.S., Figure 7B).

#### 2.2.4. Cytokines in Pre- and Post-Treatment Groups Measured by ELISA

**Figure 8:**
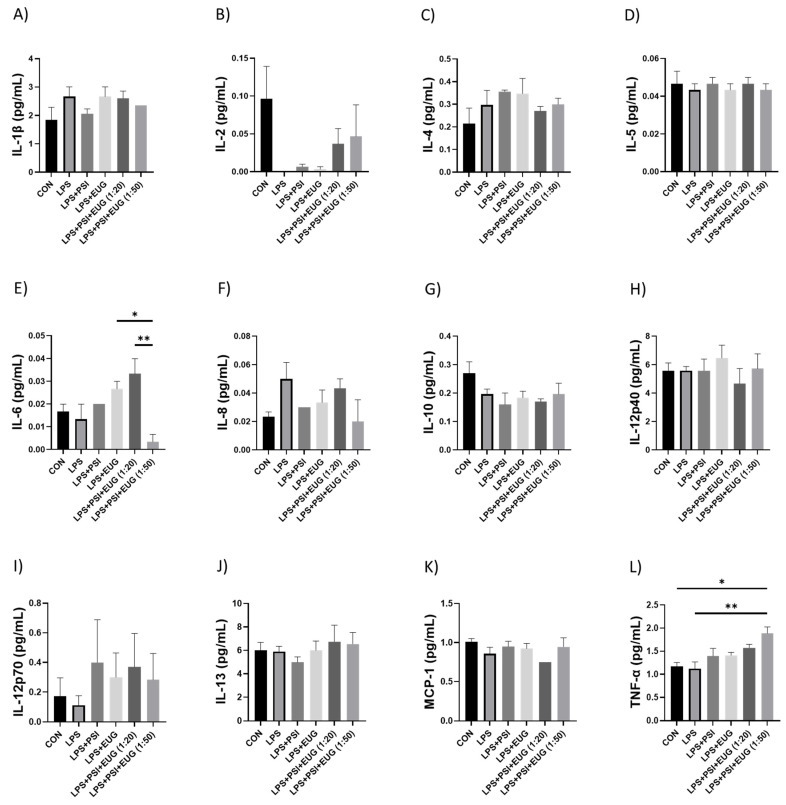
Pro-inflammatory cytokine levels in the post-treatment LPS-induced brain inflammation. The levels of (**A**) IL-1β, (**B**) IL-2, (**C**) IL-4, (**D**) IL-5, (**E**) IL-6, (**F**) IL-8, (**G**) IL-10, (**H**) IL-12p40, (**I**) IL-12p70, (**J**) IL-13, (**K**) MCP-1, and (**L**) TNF-α were measured by an ELISA. Data were analyzed with ANOVA and Tukey’s post hoc test (*n* = 3). Significance (*p*) is indicated within the figures using the following scale: *, *p* < 0.05; **, *p* < 0.01. Bars represent mean ± SEM.

Among all treatment groups, LPS + psilocybin + eugenol (1:50) demonstrated a significant decrease in expression of IL-6 compared to the LPS + psilocybin + eugenol (1:20) (Figure 8E, *p* < 0.01) and LPS + eugenol (Figure 8E, *p* < 0.05) groups. On the other hand, LPS + psilocybin + eugenol (1:50) demonstrated a significant increase in expression of TNF-α compared to the control (Figure 8L, *p* < 0.05). The same results were shown for the combined treatment group (1:50) compared to the LPS group (Figure 8L, *p* < 0.01). No other significant differences were observed (Figure 8).

**Figure 9:**
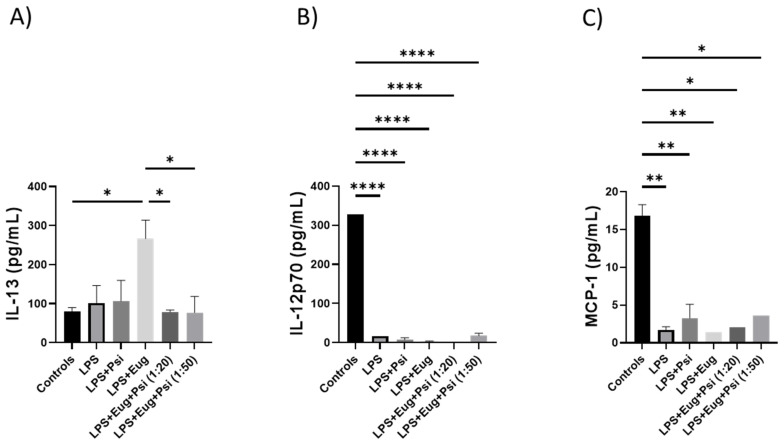
The content of pro-inflammatory cytokines in post-treatment LPS-induced inflammation in blood. The amounts of (**A**) IL-13, (**B**) IL-12p70, (**C**) MCP-1 were determined by ELISA. Data were analyzed with ANOVA and Tukey (*n* = 1–3). Significance (*p*) is indicated within the figures using the following scale: *, *p* < 0.05; **, *p* < 0.01; ****, *p* < 0.0001. Bars represent mean ± SEM.

Next, we measured pro-inflammatory cytokines collected in blood using the ELISA. As shown in Figure 9A, LPS + eugenol has a significantly higher expression of IL-13 compared to the control and LPS + Eug + Psi (1:20 and 1:50) groups (*p* < 0.05). On the other hand, the control group has significantly higher expression of IL-12p70 (*p* < 0.0001) and monocyte chemotactic protein-1 (MCP-1, *p* < 0.05) compared to all other groups (Figure 9B,C, respectively). Additional ELISA data can be found in the Supplementary Materials (Figures S12 and S13).

## 3. Discussion

The prevention and treatment of inflammation have been the main focus of many researchers. During multiple studies across several decades, we have accumulated knowledge about the role of inflammation in the development of chronic pathologies such as neurodegeneration, Alzheimer’s disease, Huntington’s disease, and Parkinson’s disease, as well as their underlying signaling pathways. In addition, the anti-inflammatory properties of natural compounds of mushrooms and plants have been the subject of interest in recent studies. In this study, we focused on psilocybin and eugenol and their effects on inflammation in the brain.

Neuroinflammatory responses have profound implications for immunity, physiological function, biochemistry, and psychological well-being. Moreover, the degree of neuroinflammation depends on the context, duration, and progression of the primary stimulus or insult. An inflammatory reaction may result in the recruitment of immune cells, edema, tissue damage, and the potential for cell death. There is, however, no universal agreement on what constitutes neuroinflammation.

In our research, we used LPS to induce inflammation in mice. LPS are among the major components of the outer membrane of Gram-negative bacteria and are widely used as inflammation-inducing agents. LPS are commonly used to model sepsis and inflammation in mammals due to their potent pro-inflammatory properties and well-studied TLR4-dependent mechanisms of activation [31,32]. One of the studies reports that administration of LPS contributes to increased neuroinflammation along with damage to the blood–brain barrier (BBB) [11]. To support that point, another study states that the level of cytokines in the central nervous system and peripheral system can be elevated by the combination of LPS and toll-like receptor 4 (TLR4), which simultaneously activates the hypothalamus–pituitary–adrenal axis. Subsequently, the release of inflammatory factors, such as IL-1β, IL-6, and TNF-α, is induced by the activation of the NF-κB signaling pathway [33].

There are many applications of the LPS model, including the study of acute lung injury, endometriosis, and acute renal injury. The use of the LPS model in neural research is also widespread [34]. We established LPS-induced inflammation models in mice, which are supported by the enhanced expression of pro-inflammatory cytokines in the brain. Afterwards, we demonstrated the effect of administrating different treatments of eugenol and psilocybin, as well as their combinations, prior to and after inflammation induction.

The anti-inflammatory properties of psilocybin and psychedelic mushrooms have been the object of recent studies. Evidence suggests that psychedelics could be useful for treating the behavioral and psychological symptoms of dementia. By upregulating neurotrophic factors, they enhance neuronal survival, promote neuronal growth, and have a profound effect on immune function. The use of psychedelics may be able to modify the progression of neurodegenerative diseases [35]. In 2020, Nkadimeng et al. investigated the antioxidant and anti-inflammatory properties of *Psilocybe natalensis* on LPS-stimulated RAW 264.7 macrophages [21]. The mushroom extracts decreased LPS-induced TNF-α and inhibited pro-inflammatory cytokines IL-1β and IL-10. The reduction in these pro-inflammatory cytokines is associated with improved health outcomes in chronic inflammation [36]. Additionally, the extracts decreased LPS-induced TNF-α and inhibited pro-inflammatory cytokines IL-1β and IL-10. In chronic inflammation, reducing these pro-inflammatory cytokines is associated with improved health outcomes [21]. They also showed that mushroom extracts contained components, such as n-hexadecanoic acid, 4h-pyran-4-one, 2,3-dihydro-3,5-dihydroxy-6-methyl, 3-octanone, and dibutyl phthalate, can induce natural anti-inflammatory and antioxidant effects. Additionally, LPS-induced nitric oxide and prostaglandin E2 production, which plays a role in inflammatory diseases, were inhibited by extracts of *Psilocybe natalensis*. Psychedelics were shown to be potent 5-HT2A receptor antagonists and to have anti-inflammatory effects via inhibition of *TNF-α* induced inflammation [22] and several downstream markers, such as *IL-6*, *IL-5*, *IL-1β*, *IL-13*, *GM-CSF*, and *MCP-1* [37]. IL-5 is a homodimeric cytokine, and its most important role lies in the differentiation, growth, activation, and survival of eosinophils as well as their recruitment to airways [38]. IL-4 and IL-13 are members of the Th2-type cytokines and play a critical role in the type II inflammatory response triggered by allergy or parasite infection. They stimulate B cell proliferation and activation of eosinophils, basophils, and mast cells. Additionally, IL-4 and IL-13 participate in fibrosis of skin and internal organs. These cytokines can switch immunoglobulin (Ig) class of IgE and IgG4 [39,40,41].

Nkadimeng et al. conducted further research in 2021 on the anti-inflammatory effects of four psilocybin-containing mushrooms on LPS-induced inflammation in human macrophage cells in vitro. *Cyclooxygenase-2* (*COX-2*) is an inducible early response gene, activated in response to various stimuli, such as LPS, IL-1, and TNF-α. The COX-2 enzyme is able to synthesize pro-inflammatory mediators, prostaglandins, which have been reported to function as immuno-suppressors. Upregulation of COX-2 has been shown to be associated with inflammation [42]. LPS stimulation significantly increased the content of COX-2, TNF-α, IL-1β, and IL-6. Treatment with the extracts reversed the LPS-induced increase in COX-2, TNF-α, IL-1β, and IL-6 in a dose-dependent manner. Researchers found that the extracts contained anti-inflammatory properties similar to those of quercetin, a well-known antioxidant flavanol found in a variety of fruits and plants [36]. Psilocybin’s role in inflammation has yet to be studied in full, and more research is needed to elucidate its therapeutic potential.

The effects of eugenol on inflammation have been studied for longer compared to psilocybin. As such, it is known that eugenol has an anti-inflammatory effect on acute lung injury induced by LPS. Eugenol pre-treatment downregulates the expression of pro-inflammatory cytokines *IL-6* and *TNF-α* and the signaling of inflammatory enzyme markers such as *COX-2* and *NF-κB*, which lead to inflammatory response inhibition [43]. Moreover, eugenol can be used as a damage-preventing agent from oxidative stress [44]. Another study reports that eugenol’s anti-apoptotic and anti-inflammatory effects can mediate the side effects of gemcitabine by increasing the activity of caspase-3 and reducing *COX-2* and *IL-1β* gene expression [45]. That study was conducted with HeLa cells, a human cervical cancer line. However, there are no studies describing the effect of combined treatment involving both eugenol and psilocybin.

After establishing the LPS-induced inflammation model in male mice, we administered psilocybin and eugenol as a pre-treatment or as a post-treatment. IL-1β is a pro-inflammatory cytokine that is involved in the regulation of pain, inflammation, homeostasis, and autoimmune conditions. It promotes the differentiation of monocytes into conventional dendritic cells [46,47]. TNF-α is a multifunctional cytokine that participates in the regulation of immune–inflammatory reactions involved in host defense against infectious, autoimmune, and endocrine diseases and cancer, and its actions help determine the survival or death of various cells [48]. Similar to IL-10, it has a pleiotropic effect on inflammation and immune response. Due to the trans-signaling mechanism of IL-6, it has a wide range of target cells, making it a key cytokine in inflammation [49]. The effect of psilocybin alone in the pre-treatment group showed higher expression of *TNF-α* and *IL-1β*, but not *IL-6* and *COX-2*, relative to the control group (Figure 4). The results of RT-qPCR (Figure 4) correlate with the results of Western blotting, which demonstrated elevated content of IL-1β and COX-2 compared to the control group (Figure 6). On the other hand, RT-qPCR results demonstrated that administrating psilocybin post-treatment resulted in a notable reduction in expression of *IL-6*, and *TNF-α*, shown in Figure 5. 

The main function of MCP-1 is to recruit monocytes and macrophages to sites of inflammation. It has also been found that MCP-1 can increase the expression of both TNF-α and IL-β [50]. IL-8 plays an important role in inflammation and wound healing and can recruit T cells and nonspecific inflammatory cells into sites of inflammation by activating neutrophils. IL-8 is mainly secreted from leukocytes and endothelial cells under special conditions such as exposure to IL-1 or TNF-α. IL-8 is chemotactic for fibroblasts; it accelerates their migration, and stimulates the deposition of tenascin, fibronectin, and collagen I during wound healing in vivo [38,39]. ELISA results, compared to the control group, demonstrated an increase in the amount of IL-6, IL-12p70, and TNF-α (Figure 8E,I,L, respectively), and a decrease in IL-2 and IL-10, (Figure 8B,G, respectively). Apart from that, post-treatment did not appear to affect the expression of IL-1β, IL-4, IL-5, IL-8, IL-10, IL-12p40, IL-13, and MCP-1 (Figure 8A,C,D,F,G,H,J,K, respectively). IL-2 cytokine is involved in the activation and regulation of the immune response. It is produced by T cells, specifically CD4+ helper cells. It takes part in effector T-cell differentiation and provides T cells with a long-lasting competitive advantage, resulting in the optimal survival and function of memory cells. Not only can IL-2 induce the proliferation of T cells and T-helper 1 and Th2 effector, but it is also crucial in the development of T memory cells [51,52]. IL-10, a cytokine, can affect the activity of multiple cell types. It inhibits the production of pro-inflammatory cytokines by inhibiting T-helper 1 (Th1) lymphocytes and stimulating B lymphocytes and Th2 lymphocytes. This leads to the downregulation of the inflammatory response [53]. IL-12p40 is a subunit of IL-12 and acts as a chemoattractant for macrophages; it also promotes the migration of bacterially stimulated dendritic cells. IL-12p40 is associated with several pathogenic inflammatory responses such as silicosis, graft rejection, and asthma. However, it is protective in a mycobacterial model [54]. Another subunit of IL-2 is IL-12p70, a pro-inflammatory cytokine composed of p35 and p40. It enhances Th1, cytotoxic CD8+ T, and NK cell responses by increasing IFN-γ production. IL-12p70 also promotes the proliferation of IL-2-dependent T cells and enhances the expression of CD25 on CD4+ Th1 cells [55]. However, despite psilocybin showing better results in post-treatment groups, psilocybin alone may not be enough to be used as an anti-inflammatory agent.

We observed changes in inflammatory markers in pre- and post-treatment eugenol groups. After using eugenol as a pre-treatment, RT-qPCR showed no significant difference between LPS and either eugenol or psilocybin pre-treatment groups, except for lower expression of *COX-2* and *TNF-α* (Figure 4C,D). The expression of COX-2 (Figure 6A) in Western blot supports results for the RT-qPCR in the pre-treatment. On the other hand, the amount of IL-1β was similar for both psilocybin and eugenol pre-treatment groups (Figure 6B). However, these results do not align with previously described studies of eugenol’s anti-inflammatory effects in the pre-treatment group. Interestingly, the post-treatment results of RT-qPCR for eugenol showed significant downregulation of *IL-6* and *TNF-α*, while no significant differences in *COX-2* or *IL-1β* expression compared to the LPS group (Figure 5A–D). The ELISA results also showed no difference between psilocybin and eugenol groups, except for slightly lower content of *IL-12p70* (Figure 8I). The cause of these results needs further investigation.

The effect of combined treatment of eugenol + psilocybin varied not only in pre- and post-treatment but depended on different ratios of these two drugs. Pre-treated mice with psilocybin + eugenol in a ratio of 1:50 demonstrated the best results compared to all other groups with no significant decrease of *IL-1β* (Figure 4A). The Western blots, however, showed a slight decrease in COX-2 and IL-1β (*p* = N.S., Figure 6A,B). Besides that, LPS + psilocybin + eugenol (1:50) demonstrated a significant decrease in IL-6 expression (Figure 8E) but not IL-1*β*. This may be due to eugenol’s influence, as similar results were described for eugenol in one of the studies [56].

## 4. Materials and Methods

### 4.1. Animals

For this study, we used C57BL/6J mice (Charles River Laboratories, Laval, QC, Canada) in accordance with the Guide to Care and Use of Animals of the Canadian Council of Animal Care, which was approved by the Animal Care Committee at the University of Lethbridge, AB, Canada.

Our study was conducted in 2 parts: part 1, investigating the effect of the i.p. injection of lipopolysaccharides (LPS) on inflammation, and part 2, investigating how treatment with psilocybin, eugenol, and their combination affects inflammation.

### 4.2. Animal Handling

For the 1st part, 8–10 week-old mice were assigned into 4 groups: 0, 4, 24, and 48 h. Mice received either an i.p. LPS injection at a dose of 0.83 mg/kg or a saline solution as a vehicle. The number of hours refers to the time between injection and tissue harvesting for each group. Mice were weighed daily. After each time point, mice were anesthetized with isoflurane. Each mouse was decapitated using the mouse guillotine. The brains were extracted, cut, washed in 1× PBS, and placed in 1.5 mL microtubes. All tissues after collection were frozen using liquid nitrogen or dry ice and stored at −80 degrees Celsius until utilized for molecular analysis.

For part 2, 8–10-week-old mice were assigned to 2 major groups: the 1st group received a pre-treatment and then LPS, while the 2nd group received LPS and then a post-treatment. Pre- and post-treatments were distributed to mice via gavage. Group 1 mice received treatments at 2 time points, 48 and 24 h before LPS injection, and tissues were harvested 24 h post-LPS treatment. Group 2 received treatments 20 h after LPS injection, and tissues were harvested 4 h later.

### 4.3. Chemicals and Apparatus

The dose of psilocybin (CAS No. 520-52-50, Applied Pharmaceutical Innovation, Edmonton, AB, Canada) used was calculated from the common dose used in humans (5 mg, based on the average weight of 70 kg) and prorated to mice using a factor of 12.3 [57]. For the combination with eugenol (CAS No. 97-53-0, Sigma-Aldrich, Saint Louis, MI, USA), three different ratios were used: 1:10, 1:20, and 1:50. LPS–L-4391-1MG, serotype 0111:B4 (Lot No. 059M4173V, SIGMA Life Science, Rehovot, Israel) was administered via intraperitoneal injection at a concentration of 0.83 mg/kg.

Pre-treatments included:

1. Control—vehicle;
2. Psilocybin (0.88 mg/kg);
3. Eugenol (17.6 mg/kg);
4. Psilocybin + eugenol (1:20; 0.88 mg/kg psilocybin and 17.59 mg/kg eugenol);
5. LPS (0.83 mg/kg);
6. Psilocybin (0.88 mg/kg) + LPS;
7. Eugenol (17.6 mg/kg) + LPS;
8. Psilocybin + eugenol (1:10; 0.88 mg/kg psilocybin and 8.8 mg/kg eugenol) + LPS;
9. Psilocybin + eugenol (1:20; 0.88 mg/kg psilocybin and 17.6 mg/kg eugenol) + LPS;
10. Psilocybin + eugenol (1:50; 0.88 mg/kg psilocybin and 44.0 mg/kg eugenol) + LPS.

Post-treatments included:

1. Vehicle;
2. LPS (0.83 mg/kg);
3. LPS + psilocybin (0.88 mg/kg);
4. LPS + eugenol (17.6 mg/kg);
5. LPS + psilocybin + eugenol (1:10);
6. LPS + psilocybin + eugenol (1:20);
7. LPS + psilocybin + eugenol (1:50).

The remaining 4 mice did not receive any treatment or gavage and acted as a control without receiving the vehicle.

Additional equipment and supplies used:

- NanoDrop 2000/2000c Spectrophotometer (Thermo Fisher Scientific, Wilmington, DE, USA).
- ECL Prime Western Blotting System (Cat No. GERPN2232, GE Healthcare, Chicago, IL, USA).
- TRIzol^®^ Reagent (Cat No. 15596018, Invitrogen, Carlsbad, CA, USA).
- FluorChem HD2 Imaging System (Cell Biosciences, Santa Clara, CA, USA).
- iScriptTM Select cDNA synthesis kit (Cat No. 1708897, BioRad, Hercules, CA, USA).
- SsoFastTM EvaGreen^®^ Supermix (Cat No. 1725202, BioRad, Hercules, CA, USA).
- C1000TM Thermal Cycler equipped with a CFX96 Touch™ Real-Time PCR Detection System (BioRad, Hercules, CA, USA).

### 4.4. Protein Extraction and Quantification

The brain tissue was crushed by pestle with 400 μL of RIPA lysis buffer in 1.5 mL microtubes. The mixture was homogenized on the shaker with Zirconium beads (Cat No. D1032-15, Cole-Parmer, QC, Canada) 3 times for 3 min with 2 min on ice in between sets. Next, 200 μL of RIPA lysis buffer was added to each microtube and put on the shaker at 4 °C for 2 h. The microtubes were then centrifuged for 10 min at 13,500 rpm. The supernatant was collected. Using the Bradford protein assay with bovine serum albumin as the standard, protein concentrations were determined via a NanoDrop 2000/2000c Spectrophotometer (Thermo Fisher Scientific, Wilmington, DE, USA).

### 4.5. Western Immunoblotting

Western immunoblotting was conducted with 150 μg/μL of protein. Samples were prepared with 4× loading buffer (0.0625 M Tris, 2% SDS, 10% glycerol, 0.01% bromophenol blue, and 1% 2-mercaptoethanol) and RIPA lysis buffer and heated at 95 °C for 5 min. The protein sample and PageRuler Plus Prestained Protein Ladder (Cat No. 26620, Thermo Scientific, MA, USA) were loaded and electrophoretically separated by SDS-PAGE into gels with a combination of 8% (top) and 12% (bottom) polyacrylamide at 60 V for 30 min and then switched to 75 V for 1 h. Polyvinylidene difluoride membranes (Amersham Biosciences, Baie d’Urfé, QC, Canada) were used to transfer resolved proteins. Then, membranes were incubated for 1 h in a blocking solution (5% skim milk in PBS, 0.5% Tween 20) at room temperature and incubated with primary antibodies COX2, IL-1β and GAPDH at 4 °C overnight.

After incubation, the membranes were washed three times with 0.1% Tween-20 in PBS (PBS-T). The membranes were incubated with 1:5000 dilution of either bovine anti-mouse secondary antibodies or donkey anti-rabbit secondary antibodies for two hours at room temperature.

Next, membranes were washed 3 times with PBS-T and then exposed to the ECL Prime Western Blotting System (Cat No. GERPN2232, GE Healthcare, Chicago, IL, USA). Chemiluminescence was detected using the FluorChem HD2 Imaging System (Cell Biosciences, Santa Clara, CA, USA). Unaltered PVDF membranes were stained with Coomassie blue (BioRad, Hercules, CA, USA) to confirm equal protein loading. Signals were quantified using the NIH Image J64 software and normalized relative to GAPDH or Coomassie staining as indicated.

### 4.6. RNA Isolation

RNA was isolated from brain tissue using TRIzol^®^ Reagent (Invitrogen, Carlsbad, CA, USA), purified using an RNAesy kit (Qiagen), according to the manufacturer’s instructions, and quantified using a NanoDrop 2000c (Thermo Fisher Scientific, Wilmington, DE, USA).

### 4.7. Quantitative Real-Time PCR (qRT-PCR)

Quantitative real-time PCR (qRT-PCR) was performed on brain tissue from all experimental groups. According to the manufacturer’s instructions, cDNA was generated with 500 ng RNA using the iScriptTM Select cDNA synthesis kit (Cat No. 1708897, BioRad, Hercules, CA, USA). PCR reactions were based on the SsoFastTM EvaGreen^®^ Supermix (Cat No. 1725202, BioRad, Hercules, CA, USA) and 500 nM of forward and reverse primers specific for target sequences of interest. Primers were designed using the https://www.idtdna.com/Primerquest platform, accessed on 2 June 2022. Primers were checked before on dilution series of normal brain tissue cDNA. The reference gene (*GAPDH*) was analyzed with the GeNorm method. The reactions were analyzed on a C1000TM Thermal Cycler equipped with a CFX96 Touch™ Real-Time PCR Detection System (BioRad, Hercules, CA, USA). The PCR programs were run according to the SsoFastTM guidelines with annealing temperatures as specified for the specific primer pairs. Expression analysis was performed with the BioRad Software (CFX Manager) and was based on the ΔΔCt method with the reference genes that were stably expressed in the GeNorm analysis. Each experiment included three biological replicates for each group and two technical replicates per sample. The genes used for qRT-PCR were *IL-1β*, *TNF-α*, *IL6*, *COX2*, and *Amylase A* (Figure S1).

### 4.8. Enzyme-Linked Immunoassay (ELISA)

Samples for the ELISA were prepared using extracted proteins. Three samples from each post-treatment group, except for the psilocybin + eugenol group, were selected randomly, for a total of 18 samples. After thawing, protein samples were centrifuged for 10 min at 5000 rpm. Supernatant was aliquoted into 0.2 μL labeled microtubes, put in a box, and sent to Eve Technologies (Calgary, AB, Canada) for the enzyme-linked immunoassay. All procedures were carried out on ice.

### 4.9. Statistical Analysis

Data were analyzed using GraphPad Prism 9 (GraphPad Software, San Diego, CA, USA) and are presented as means with standard error of the mean (SEM) error bars. Multiple unpaired Student’s *t*-tests with a false discovery rate correction (Q = 5%) were used for comparisons between two groups. A one-way analysis of variance (ANOVA) followed by Dunnett’s or Tukey’s post hoc test were used for the analysis of three and more groups. *p* values < 0.05 were considered statistically significant.

## 5. Conclusions

This study established a model of LPS-induced inflammation in the brains of male mice and demonstrated the effects of treatment with eugenol, psilocybin, and their combination on the expression of pro-inflammatory cytokines. In addition, our study demonstrated the anti-inflammatory effects of combined treatment with psilocybin and eugenol in brain tissue, which have not previously been described. With growing interest in psilocybin applications for medical purposes, this study provides useful insights into its effect on inflammation, which will help guide future research in this area.

## Data Availability

Data available on request.

## Supplementary Materials

The following supporting information can be downloaded at: https://www.mdpi.com/article/10.3390/molecules28062624/s1, Figure S1: The effect of LPS i.p. injections on mRNA expression in brain tissue in Figure 1; Figure S2: The effect of vehicle treatments on expression of COX2, IL-1β, GAPDH in 4, 24, and 48 h time points in Figure 2; Figure S3: Original Western blots of brain tissue proteins showing COX-2 (molecular weight is 72 kDa) in Figure 2; Figure S4: Original Western blots of brain tissue proteins showing IL-1β (molecular weight is 17 kDa) in Figure 2; Figure S5: Original Western blots of brain tissue proteins showing GAPDH (molecular weight is 36 kDa) in Figure 2; Figure S6: Original Western blots of brain tissue proteins showing COX-2 (molecular weight is 72 kDa) in Figure 6; Figure S7: Original Western blots of brain tissue proteins showing IL-1β (molecular weight is 17 kDa) in Figure 6; Figure S8: Original Western blots of brain tissue proteins showing GAPDH (molecular weight is 36 kDa) in Figure 6; Figure S9: Original Western blots of brain tissue proteins showing COX-2 (molecular weight is 72 kDa) in Figure 7; Figure S10: Original Western blots of brain tissue proteins showing IL-1β (molecular weight is 17 kDa) in Figure 7; Figure S11: Original Western blots of brain tissue proteins showing GAPDH (molecular weight is 36 kDa) in Figure 7; Figure S12: The content of inflammatory cytokines in post-treatment LPS-induced inflammation in brain tissue in Figure 8; Figure S13: The content of pro-inflammatory cytokines in post-treatment LPS-induced inflammation in blood in Figure 9; Table S1: Antibodies used for Western blots; Table S2: Primer sequences for qPCR analysis; Table S3: Significance Matrix for Body Weight Change in Figure 3.

## Sample Availability

Samples of the compounds can be obtained from Applied Pharmaceutical Innovation and Sigma-Aldrich.
