# Supplementary material for: The Effect of Combined Treatment of Psilocybin and Eugenol on Lipopolysaccharide-Induced Brain Inflammation in Mice
Source: Molecules. 2023 Mar 14;28(6):2624. doi: 10.3390/molecules28062624 (PMC10056123; doi:10.3390/molecules28062624)
Supplement: Supplementary file 1 [file molecules-28-02624-s001.zip › molecules-2242576-supplementary.pdf]

Supplementary Materials

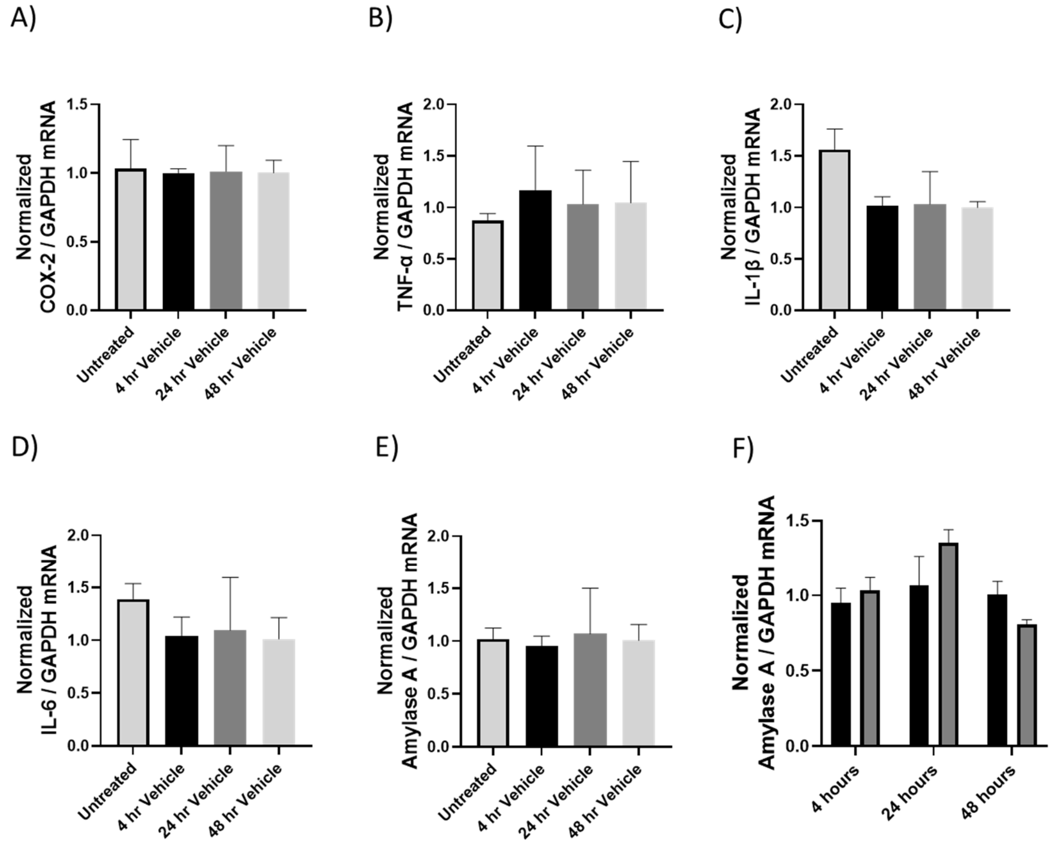

Figure S1. The effect of LPS i.p. injections on mRNA expression in brain tissue in Figure 1. Changes of mRNA expression as measured by RT-qPCR for: (**A**) COX-2 (**B**) TNF*α* (C) *IL-1β* (**D**) *IL-6* (**E, F**) *Amylase A*. Glyceraldehyde-3-phosphate dehydrogenase (*GAPDH*) was used as a loading control. Data were analyzed with (**A-E**): Multiple t-tests (FDR: Q=5%, n=5-6) and (**F**): t-test (n=3-6). Bars represents Mean ± SEM

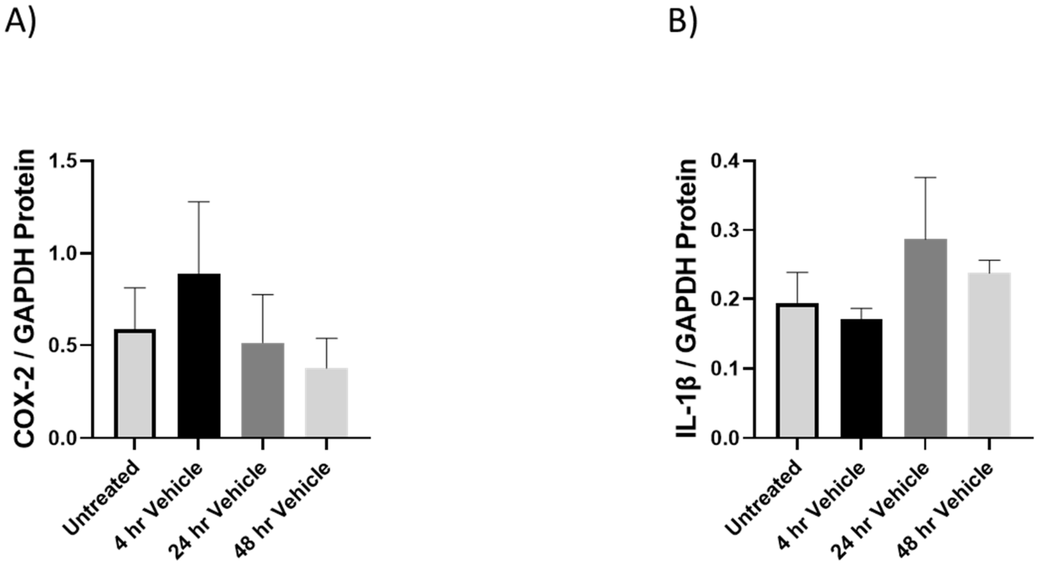

Figure S2. The effect of vehicle treatments on expression of COX2, IL-1β, GAPDH in 4h, 24h and 48h time points in Figure 2. Figures represents changed protein expression for selected genes measured by Western blot. Glyceraldehyde-3-phosphate dehydrogenase (GAPDH) was used as a loading control. Relative densitometry was presented as a ratio of target protein to GAPDH. (A) COX 2 expression (B) IL-1β expression. (A-B): ANOVA / Tukey (n=3). Bars represent Mean ± SEM.

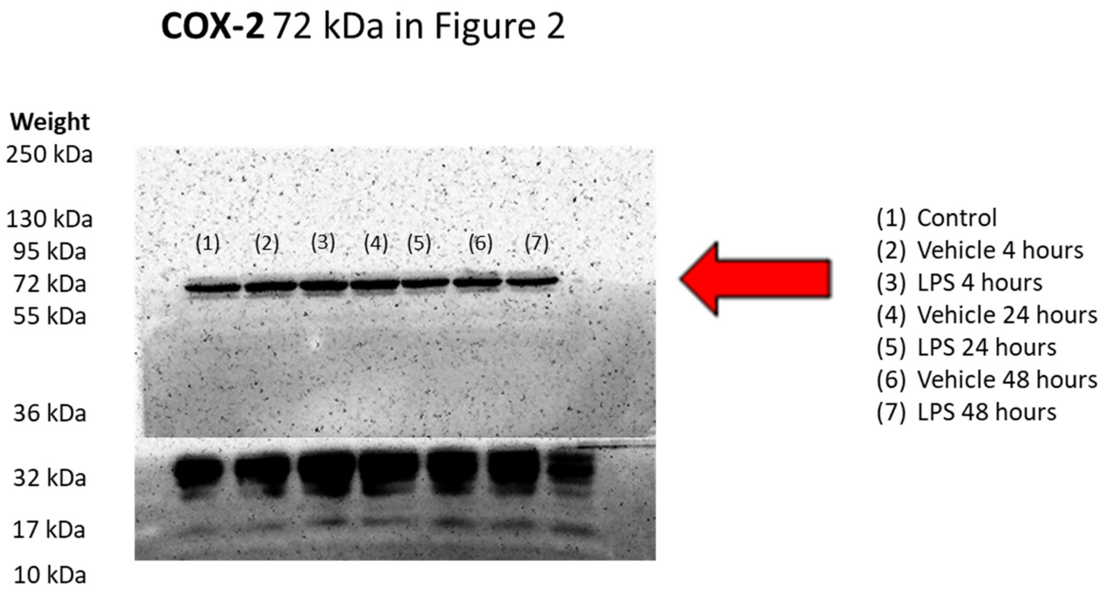

Figure S3. Original Western blots of brain tissue proteins showing COX-2 (molecular weight is 72 kDa). Red arrow indicates bands shown in Figure 2. This blot membrane has been cut in 2 pieces. Each piece received different exposure time and images has been taken separately.

IL-1β 17 kDa in Figure 2

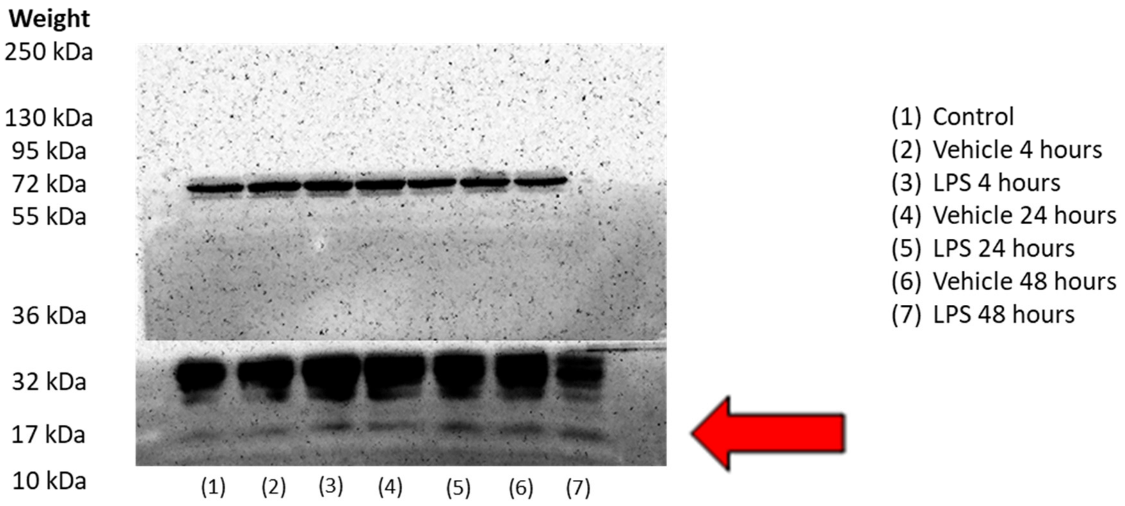

Figure S4. Original Western blots of brain tissue proteins showing IL-1β (molecular weight is 17 kDa). Red arrow indicates bands shown in Figure 2. This blot membrane has been cut in 2 pieces. Each piece received different exposure time and images has been taken separately.

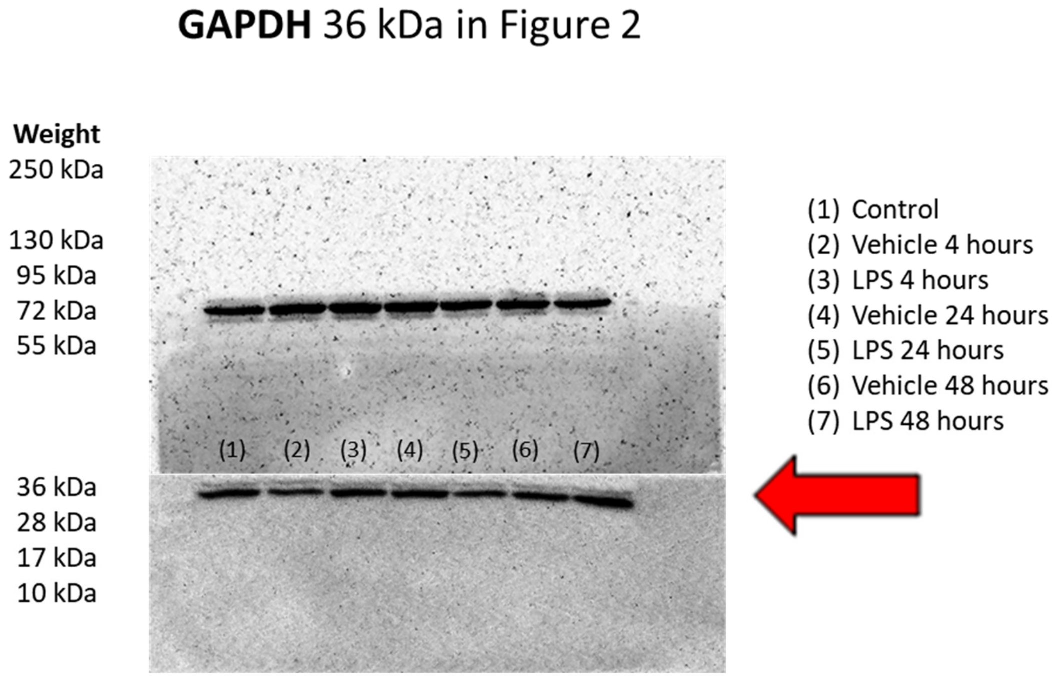

Figure S5. Original Western blots of brain tissue proteins showing GAPDH (molecular weight is 36 kDa). Red arrow indicates bands shown in Figure 2. This blot membrane has been cut in 2 pieces. Each piece received different exposure time and images has been taken separately. Bottom piece has been re-blotted and re-incubated with GAPDH.

COX-2 72 kDa in Figure 6

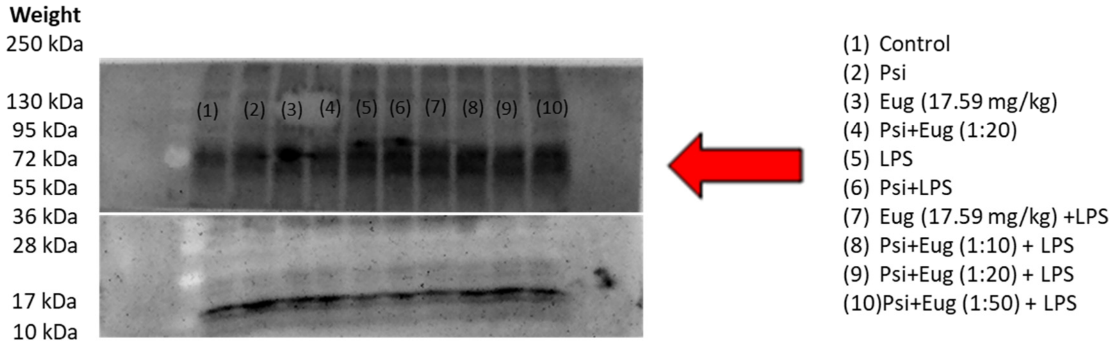

Figure S6. Original Western blots of brain tissue proteins showing COX-2 (molecular weight is 72 kDa). Red arrow indicates bands shown in Figure 6. This blot membrane has been cut in 2 pieces. Each piece received different exposure time and images has been taken separately.

IL-1β 17 kDa in Figure 6

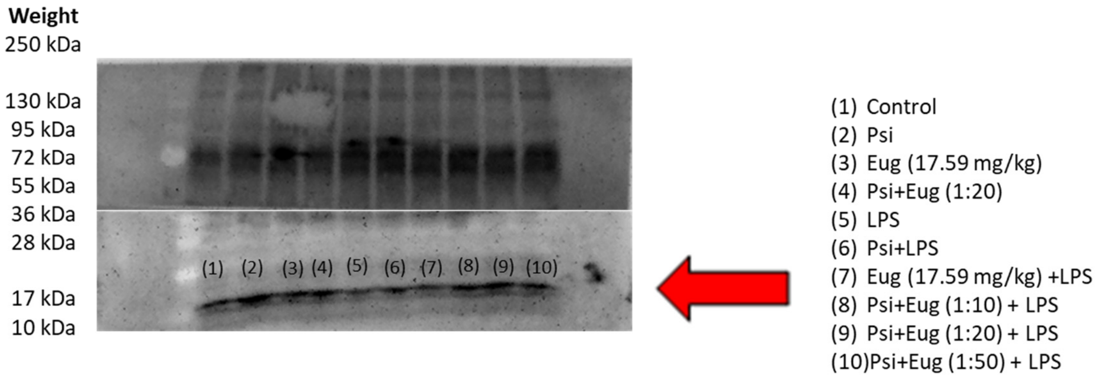

Figure S7. Original Western blots of brain tissue proteins showing IL-1β (molecular weight is 17 kDa). Red arrow indicates bands shown in Figure 6. This blot membrane has been cut in 2 pieces. Each piece received different exposure time and images has been taken separately.

GAPDH 36 kDa in Figure 6

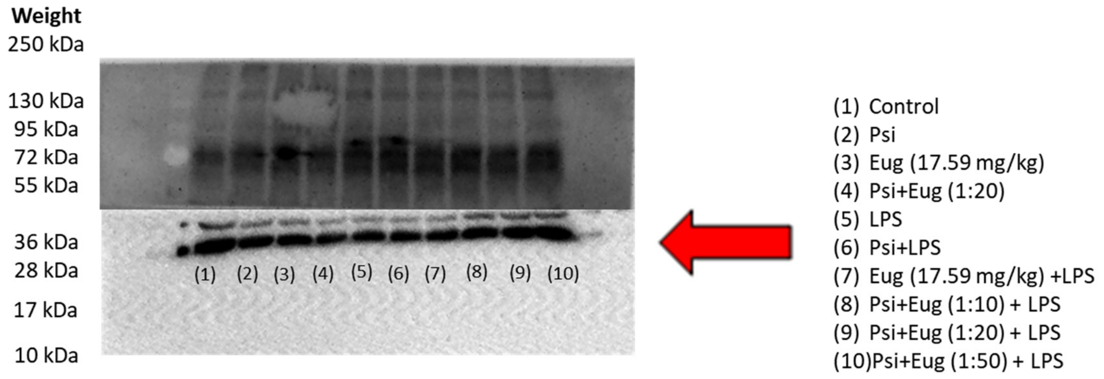

Figure S8. Original Western blots of brain tissue proteins showing GAPDH (molecular weight is 36 kDa). Red arrow indicates bands shown in Figure 6. This blot membrane has been cut in 2 pieces. Each piece received different exposure time and images has been taken separately. Bottom piece has been re-blotted and re-incubated with GAPDH.

COX-2 72 kDa in Figure 7

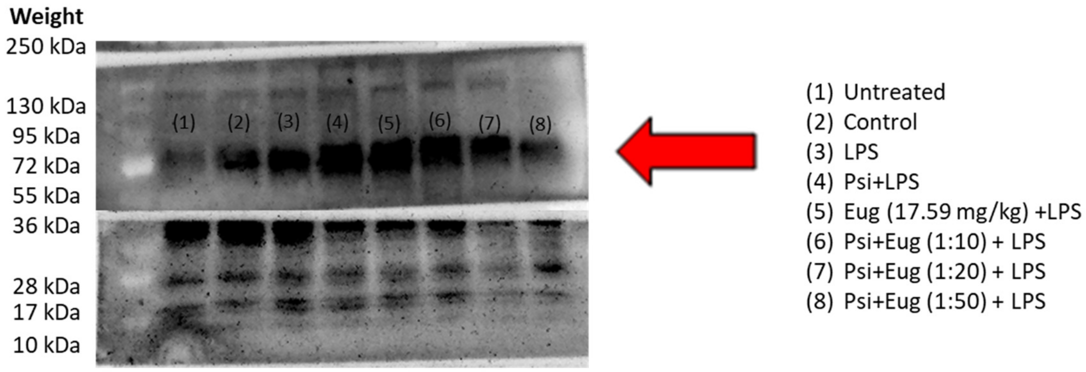

Figure S9. Original Western blots of brain tissue proteins showing COX-2 (molecular weight is 72 kDa). Red arrow indicates bands shown in Figure 7. This blot membrane has been cut in 2 pieces. Each piece received different exposure time and images has been taken separately.

IL-1β 17 kDa in Figure 7

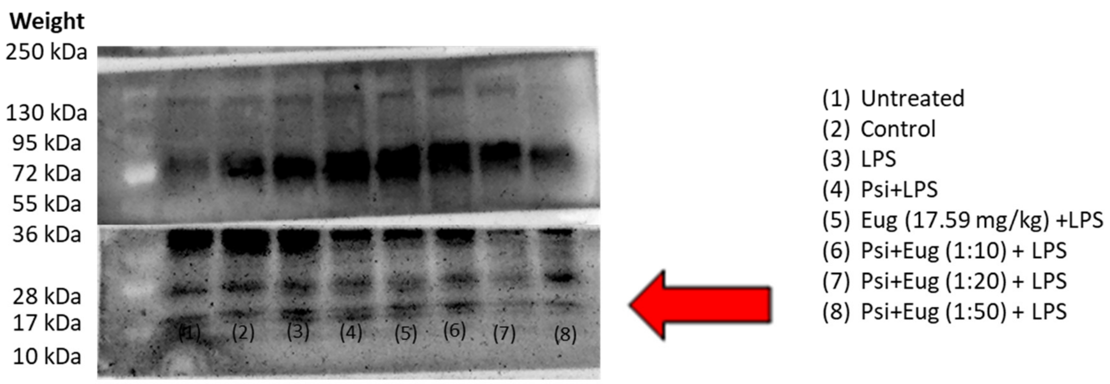

Figure S10. Original Western blots of brain tissue proteins showing IL-1β (molecular weight is 17 kDa). Red arrow indicates bands shown in Figure 7. This blot membrane has been cut in 2 pieces. Each piece received different exposure time and images has been taken separately.

GAPDH 36 kDa in Figure 7

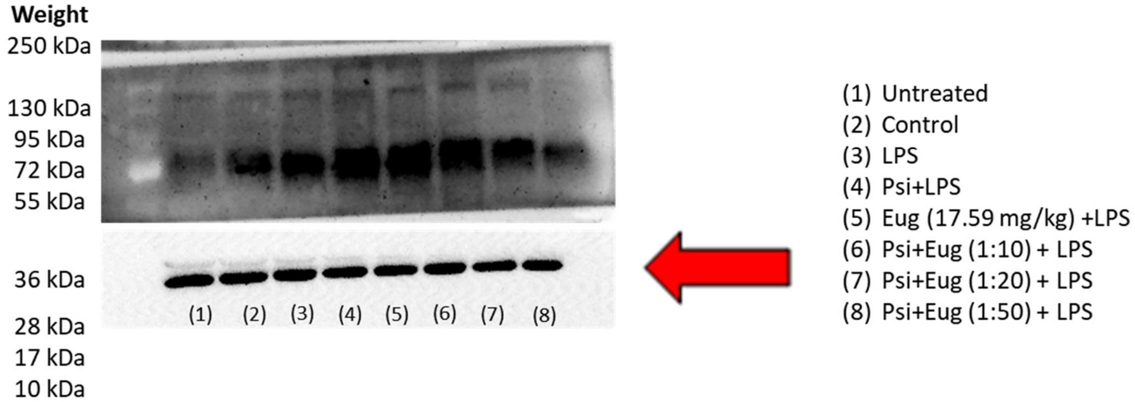

Figure S11. Original Western blots of brain tissue proteins showing GAPDH (molecular weight is 36 kDa). Red arrow indicates bands shown in Figure 7. This blot membrane has been cut in 2 pieces. Each piece received different exposure time and images has been taken separately. Bottom piece has been re-blotted and re-incubated with GAPDH.

Table S1. Antibodies used for Western blots

**Antibody**	**Supplier, Cat No**	**Dilution**
Mouse anti-GAPDH (0411)	Santa Cruz, sc-47724	1:5000 in 5% milk (PBST)
Bovine anti-Mouse	Santa Cruz, sc-2371	1:5000 in 5% milk (PBST)
Donkey anti-Rabbit	Santa Cruz, sc-2313	1:5000 in 5% milk (PBST)
COX-2 (29)	Santa Cruz, sc-19999	1:5000 in 5% milk (PBST)
IL-1β (B122)	Santa Cruz, sc-12742	1:5000 in 5% milk (PBST)

Abcam, Abcam Inc, Cambridge, United Kingdom; **BSA**, Bovine Serum Albumin; **PBST**, 1x Phosphate-Buffered Saline, 0.1 % Tween® 20; **Santa Cruz**, Santa Cruz Biotechnology, Inc., Texas, United States; **Cell Signaling**, Cell Signaling Technologies, Massachusetts, United States

Table S2. Primer sequences for qPCR analysis

Target Gene	Sequence Forward (5' → 3')	Sequence Reverse (5' → 3')
IL1B **ACC# NM_008361.4**	CAGGCAGGCAGTATCACTCATT	AAGAAGGTGCTCATGTCCTCATC
TNFA **ACC# NM_001278601.1**	GCCTCTTCTCATTCCTGCTTGT	TGGGAACTTCTCATCCCTTTGG
IL6 **ACC# NM_001314054.1**	GACTTCCATCCAGTTGCCTTCT	TATCCTCTGTGAAGTCTCCTCTCC
COX2 **ACC# NM_011198.4**	CCTTCTCCAACCTCTCCTACTACA	AGCTCCTTATTTCCCTTCACACC
Amylase a **ACC# nm-011198.4**	GGACTTTCCTGGAGTTCCCTATTC	CCTGAGCAGCATCTTGGTAGTT
GAPDH **ACC# XM_036165840.1**	CATCACTGCCACCCAGAAGA	AGTGGATGCAGGGATGATGTT

Table S3. Significance Matrix for Body Weight Change in Figure 3. ANOVA & Tukey (*n*=4-6) Significance (p) is indicated within the figures using the following scale: *, $p < 0.05$; **, p<0.01; ***, p<0.001;****, p<0.0001.

	Untreated	LPS + Psi + Eug (1:50)	LPS + Psi + Eug (1:20)	LPS + Psi + Eug (1:10)	LPS + EUG	LPS + Psi	Control - Post	PSI + Eug (1:50) + LPS	PSI + Eug (1:20) + LPS	PSI + Eug (1:10) + LPS	Eug + LPS	Psi + LPS	LPS	Psi + Eug (1:10)	Eug	Psi	Control - Pre
Untreated		**	*	**	**	*	ns	**	****	***	****	***	**	ns	ns	ns	ns
LPS + Psi + Eug (1:50)	**		ns	ns	ns	ns	**	ns	ns	ns	ns	ns	ns	ns	ns	ns	ns
LPS + Psi + Eug (1:20)	*	ns		ns	ns	ns	**	ns	ns	ns	ns	ns	ns	ns	ns	ns	ns
LPS + Psi + Eug (1:10)	**	ns	ns		ns	ns	***	ns	ns	ns	ns	ns	ns	ns	ns	ns	ns
LPS + EUG	**	ns	ns	ns		ns	ns	ns	ns	ns	ns	ns	ns	ns	ns	ns	ns
LPS + Psi	*	ns	ns	ns	ns		**	ns	ns	ns	ns	ns	ns	ns	ns	ns	ns
Control - Post	ns	**	**	***	ns	**		***	****	***	****	****	**	ns	ns	ns	ns
PSI + Eug (1:50) + LPS	**	ns	ns	ns	ns	ns	***		ns	ns	ns	ns	ns	ns	ns	ns	ns
PSI + Eug (1:20) + LPS	****	ns	ns	ns	ns	ns	****	ns		ns	ns	ns	ns	*	*	*	ns
PSI + Eug (1:10) + LPS	***	ns	ns	ns	ns	ns	***	ns	ns		ns	ns	ns	ns	ns	ns	ns
Eug + LPS	****	ns	ns	ns	ns	ns	****	ns	ns	ns		ns	ns	**	*	**	*
Psi + LPS	***	ns	ns	ns	ns	ns	****	ns	ns	ns	ns		ns	ns	ns	ns	ns
LPS	**	ns	ns	ns	ns	ns	**	ns	ns	ns	ns	ns		ns	ns	ns	ns
Psi + Eug (1:10)	ns	ns	ns	ns	ns	ns	ns	ns	*	ns	**	ns	ns		ns	ns	ns
Eug	ns	ns	ns	ns	ns	ns	ns	ns	*	ns	*	ns	ns	ns		ns	ns
Psi	ns	ns	ns	ns	ns	ns	ns	ns	*	ns	**	ns	ns	ns	ns		ns
Control - Pre	ns	ns	ns	ns	ns	ns	ns	ns	ns	ns	*	ns	ns	ns	ns	ns	

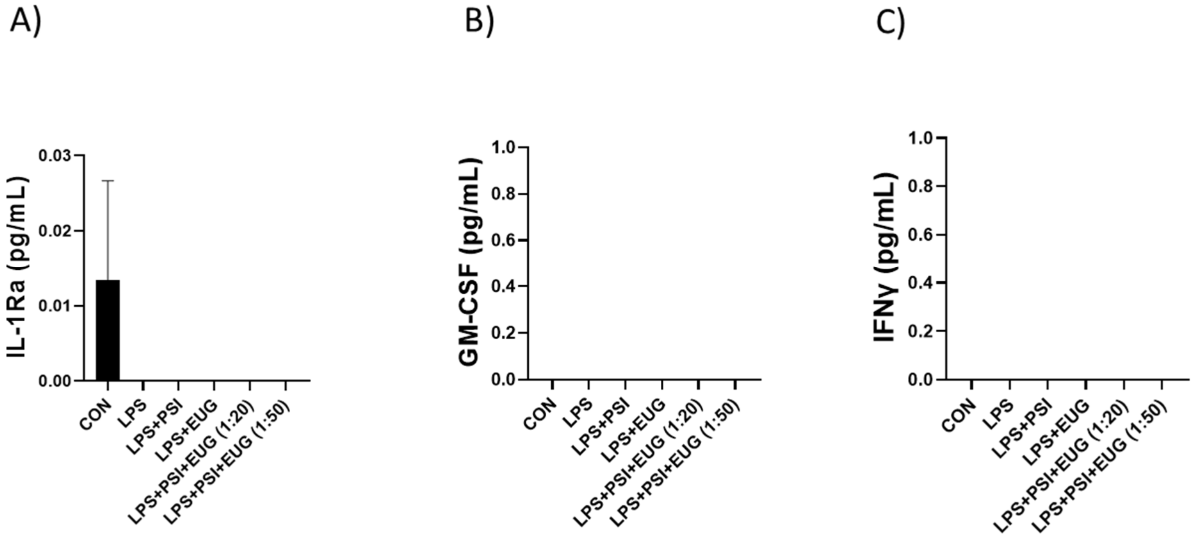

Figure S12. The content of inflammatory cytokines in post-treatment LPS-induced inflammation in brain tissue in Figure 8. The levels of (A) IL-1Ra, (B) GM-CSF, (C) IFNy. Data were analyzed with ANOVA & Tukey (*n*=3). Bars represent Mean ± SEM.

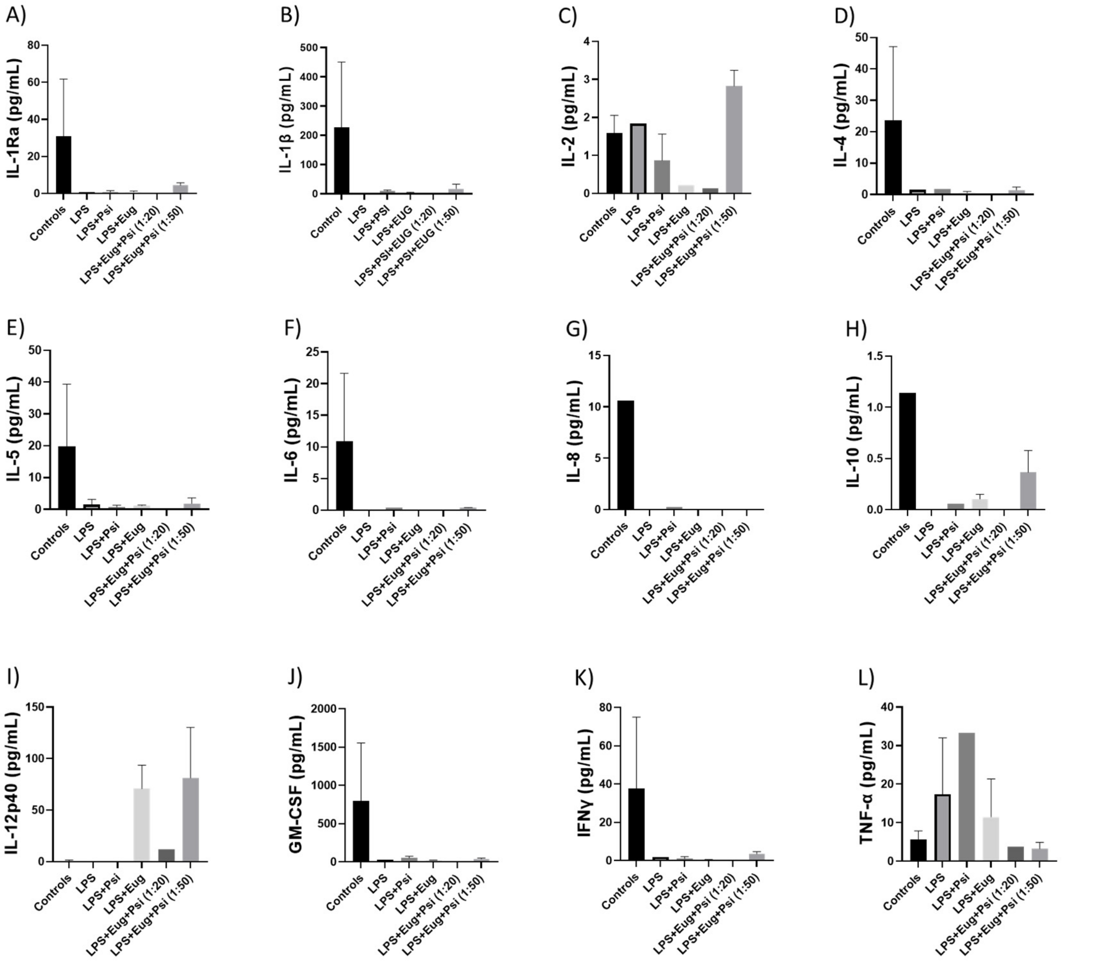

Figure S13. The content of proinflammatory cytokines in post-treatment LPS-induced inflammation in blood in Figure 9. The levels of (A) IL-1Ra, (B) IL-1β, (C) IL-2, (D) IL-4, (E) IL-5, (F) IL-6, (G) IL-8, (H) IL-10, (I) IL-12p40, (J) GM-CSF, (K) IFNγ, (L) TNFα. Data were analyzed with ANOVA & Tukey (n=1-3). Bars represent Mean ± SEM.
